# TDF Monotherapy Is Effective Regardless of Prior Nucleos(t)ide Analogue Treatment in Chronic Hepatitis B Patients in China

**DOI:** 10.1155/2017/2463197

**Published:** 2017-04-12

**Authors:** Mingxing Huang, Guoli Lin, Hong Shi, Yuankai Wu, Yusheng Jie, Zhe Zhu, Yutian Chong

**Affiliations:** ^1^Department of Infectious Diseases, The Fifth Affiliated Hospital of Sun Yat-sen University (SYSU), Zhuhai, Guangdong, China; ^2^Department of Infectious Diseases, The Third Affiliated Hospital, Sun Yat-sen University (SYSU), Guangzhou, Guangdong, China; ^3^Department of Stem Cell Biology and Regenerative Medicine, Cleveland Clinic, Lerner Research Institute, Cleveland, OH 44195, USA

## Abstract

**Background/Aims:**

Many patients had to transfer to tenofovir disoproxil fumarate (TDF) if there is other nucleos(t)ide analogue (NA) resistance. We aimed to investigate antiviral effects of TDF monotherapy between NA-naive and NA-experienced chronic hepatitis B (CHB) patients in China.

**Methods:**

A total of 102 NA-naive and NA-experienced CHB patients with TDF monotherapy (300 mg/day) were retrospectively analyzed for useful parameters up to 72 weeks.

**Results:**

There were 36 and 66 patients with matched HBV DNA baseline level in NA-naïve and NA-experienced group, respectively. There were no significant differences between NA-naïve and NA-experienced groups in HBV DNA levels (all *P* > 0.05) and HBV DNA undetectable rates (all *P* > 0.05) at all time points. At the end of follow-up, HBV DNA undetectable rates in NA-naïve and NA-experienced group were 96.2% (25/26) and 91.8% (45/49), respectively (*P* = 0.476). Baseline HBV DNA level was the only independent predictor for HBV DNA negative time (*P* = 0.018). In addition, 27.8% (5/18) and 11.4% (4/35) achieved HBeAg seroconversion at the end of the follow-up, respectively (*P* = 0.133).

**Conclusions:**

TDF monotherapy was effective regardless of prior NA experienced. Baseline HBV DNA was a key predictive factor for HBV DNA negative time in TDF monotherapy.

## 1. Introduction

Hepatitis B virus (HBV) is a major global health problem, with an estimated number of 240 million chronically infected HBV patients worldwide [1]. The risk of developing of cirrhosis and hepatocellular carcinoma (HCC) following HBV acquisition remains high. As a consequence, more than 686,000 people die every year due to complications of hepatitis B [1–4]. HBV is therefore one of the most hazardous viral pathogens for humans and a pressing global public health concern [5]. However, highly effective antiviral therapies and guidelines for screening of HBV infected patients for liver cancer are still missing.

High HBV DNA levels correlate with increased risk of HCC in chronic hepatitis B (CHB). Recently, the main treatment goal for (CHB) is to significantly suppress viral replication with the goal of preventing severe liver complications such as fibrosis and cirrhosis, liver failure, and development of hepatocellular carcinoma [6, 7]. In most cases, oral antivirus methods are used, including nucleotide/nucleoside analogues lamivudine (LMV), telbivudine (LdT), adefovir dipivoxil (ADV), entecavir (ETV), or tenofovir (TDF), but some HBV-monoinfected patients are treated with pegylatedinterferon-alpha [7]. The nucleotide and nucleoside analogues are competitive inhibitors of the HBV reverse transcriptase, as incorporation of the analogues into the DNA strand by the reverse transcriptase causes transcription termination, thereby inhibiting viral replication [8].

TDF is an oral prodrug of the nucleotide analogue tenofovir and it is a potent and selective inhibitor of HBV DNA polymerase/reverse transcriptase (pol/RT) in vitro [9]. TDF is currently approved for treatment of CHB in patients aged 12 years and older.

TDF was more effective than ADV in viral suppression and alleviating histologic inflammation, which has been showed in two international, multicenter, randomized, double-blind phase 3 studies comparing once-daily TDF and once-daily adefovir dipivoxil (ADV) [10]. In addition, at year 5 of antiviral therapy, TDF therapy led to histological improvement (defined as a ≥2-point reduction in Knodell necroinflammatory score with no worsening of fibrosis) in 87.4% (304/348) of patients, and 74.0% (71/96) had reversal of baseline cirrhosis [11]. Similar outcomes have also been reported in China in several follow-up studies [12–14].

However, there is still limited data to show whether TDF is effective for the NA-experienced and NA-naïve CHB patients in China. Here, we report the efficacy, safety, and resistance results of patients between these two groups of CHB treated patients through a follow-up study in our hospital.

## 2. Materials and Methods

### 2.1. Patient Selection

All the selected patients in our follow-up study group were from the 3rd Affiliated Hospital, Sun Yat-sen University. The study was conducted in accordance with the guidelines of the Declaration of Helsinki and was approved by the 3rd Affiliated Hospital Ethical Committee at SYSU. The study design and manuscript preparation fully followed the guideline from the STROBE statement [15]. Written informed consent was obtained from all patients.

### 2.2. Follow-Up Time

**Figure 1:**
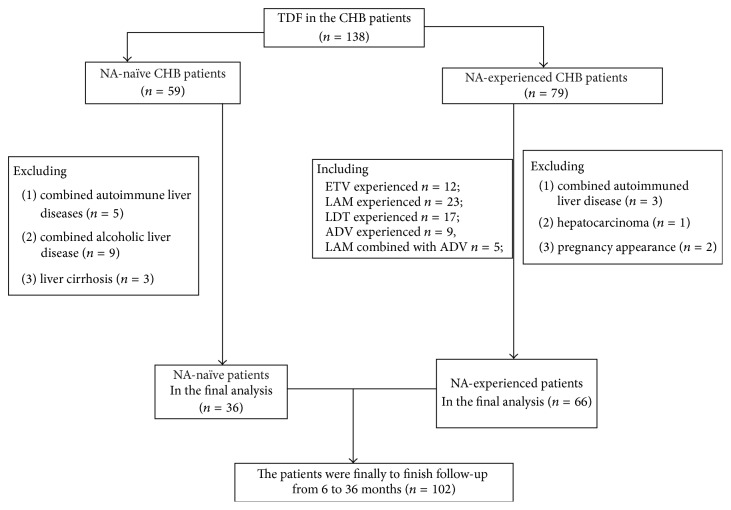
The flow chart of the patients enrolled.

From June 1, 2012, to December 31, 2015, 102 CHB were enrolled in this study, including 36 in the NA-naïve treatment group and 66 NA-experienced treatment group. Parameters such as age, height, weight, serum alanine, aminotransferase (ALT) and HBV DNA levels at baseline, gender, alcohol use, and smoking status were recorded for each patient prior to treatment. All the patients were followed up once at least every 3 months in order to collect the serum testing. All the patients corresponded to the guideline of prevention and treatment for chronic hepatitis B, which was implemented by guidelines for prevention and treatment of chronic hepatitis B (2010 edition). The demographics of the patients are shown in Table 1. Exclusion criteria were as follows: Patients were excluded from this study if they (1) were coinfected with other hepatitis viruses or suffered from comorbidities; (2) displayed alcoholic, drug-induced, or autoimmune liver diseases; (3) were pregnant or lactating females. In the present study, we retrospectively analyzed 138 NA-naïve and NA-experienced treated patients from the 3rd Affiliated Hospital, Sun Yat-sen University. Among them, 102 initially treated patients were selected since their medical records met all criteria and follow-up time requirements (Figure 1).

### 2.3. Therapeutic and Detection Methods

All the patients received daily TDF (300 mg) (Viread, GSK Co.) monotherapy. They participated in our follow-up study under their own consent. Liver and kidney functions were tested using Hitachi 7180 (Hitachi, Ltd., Tokyo, Japan) and Olympus 64 (Olympus Co., Tokyo, Japan). Normal range of ALT value is 5–35 U/L. The lower limit of serum HBV DNA detection was 100 IU/ml (Da an Genetics). The following HBV infection parameters were assessed: HBeAg and anti-HBe status, serum ALT and HBV DNA levels during 4, 12, 24, 36, 48, 60, 72, and 96 weeks of treatment, time to ALT normalization, time to undetectable HBV DNA level, and HBeAg seroconversion and total duration of follow-up. Definitions: complete viral suppression was defined as undetectable serum HBV DNA (<100 IU/mL, or below the lower limit of quantification of the PCR assay). Virological breakthrough was defined as a >1 log_10_ IU/mL increase in serum HBV DNA levels from nadir in two consecutive measurements. ALT ≤ 40 U/L was considered as normal.

### 2.4. Statistical Analyses

The SPSS 13.0 software (SPSS Inc., Chicago, IL, USA) was used for all statistical analyses. Categorical variables were defined as proportion (%) and compared by Chi-square or Fisher's exact test. Continuous variables are mean ± standard deviation (SD) and were assessed by Student's *t*-test or Mann–Whitney *U* test, as appropriate. Cox regression analysis was performed in search of variables determining the virological response. Cumulative rates of complete viral suppression were analyzed by the Kaplan-Meier method. *P* < 0.05 was considered statistically significant.

## 3. Results

### 3.1. Patient Characteristics

A total of 102 patients were included in this study, comprising 36 and 66 cases in NA-naïve group and NA-experienced group, respectively. Their average ages were 35 (26–61) years in the NA-naïve group and 34.0 (24–61) years in the NA-experienced group. There were no statistically significant differences in age, gender, height, weight, smoking and drinking history, HBV family history, baseline ALT levels, and HBV DNA levels between two groups' patients (Table 1).

### 3.2. ALT Levels Alteration and Normalization Rates

**Figure 2:**
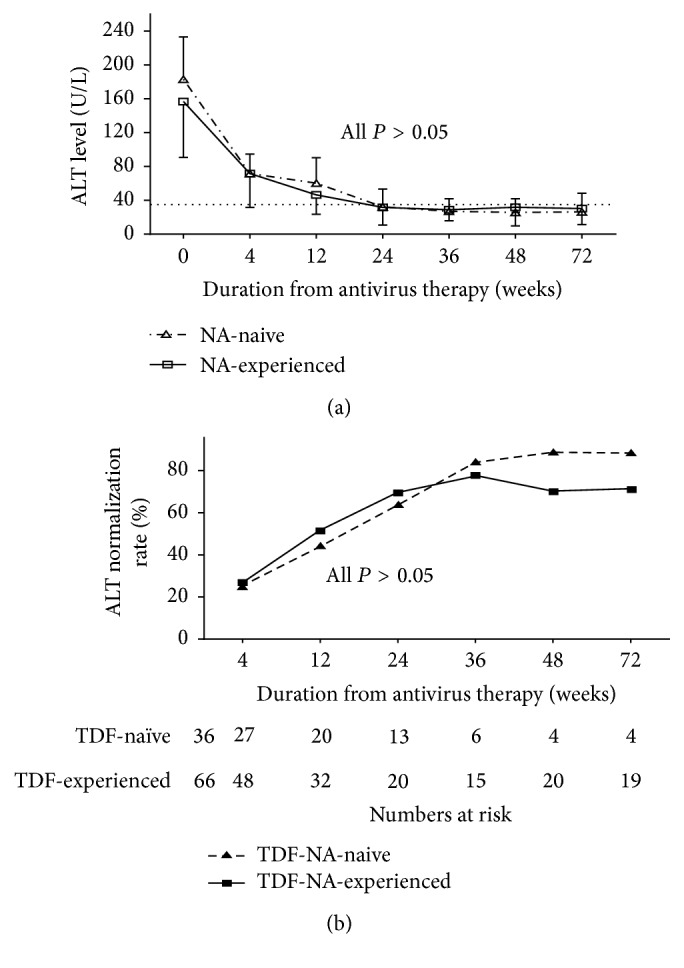
ALT levels changes (a) and the rates of ALT normalizations (b) during antivirus therapy in each group. There was no significant difference in NA-naïve and NA-experienced group neither in ALT levels changes nor in ALT normalization rates. However, ALT level decreased significantly from week 0 to week 12 in both NA-naïve and NA-experienced group. ALT: Alanine aminotransferase; TDF: tenofovir disoproxil fumarate; NA: nucleos(t)ide analogue (all *P* < 0.05).

The ALT levels progressively decreased to normal following administration of antiviral drugs treatment in both groups. ALT levels dropped from 183.0 ± 50.0 U/L at week 0 to 26.3 ± 13.0 U/L at week 72 in the NA-naïve group and from 156.8 ± 66.0 U/L at week 0 to 30.3 ± 19.0 U/L at week 72 in NA-experienced group (*P* = 0.229). No other significant differences were found at any other time point. The rates of patients with normalized serum ALT levels at weeks 4, 12, 24, 36, 48, and 72 did not differ significantly between two groups (all *P* > 0.05) (Figure 2).

### 3.3. Virological Response

**Figure 3:**
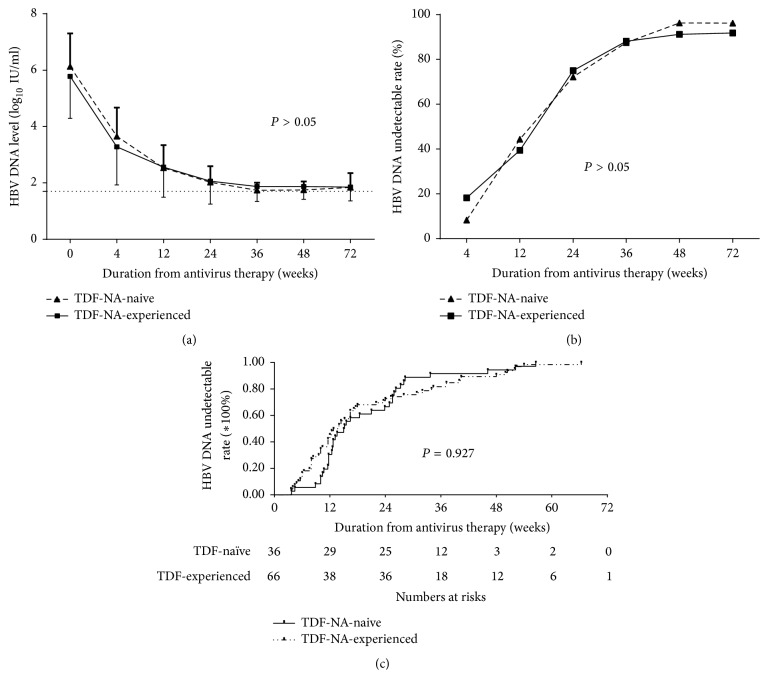
HBV DNA level (a) decreased and HBV DNA undetectable rates (b) increased during the antivirus therapy. In addition, undetectable HBV DNA cumulative undetectable rates (c) also increased but neither had significant difference in the two groups. TDF: tenofovir disoproxil fumarate; NA: nucleos(t)ide analogue.

HBV DNA levels in NA-naïve and NA-experienced groups were both decreased significantly for 72 weeks (*P* < 0.05). However, there were no significant differences between NA-naïve and NA-experienced groups at weeks 4, 12, 24, 36, 48, and 72 in HBV DNA levels (*P* values were 0.128, 0.842, 0.821, 0.121, 0.224, and 0.905, resp.). In addition, HBV DNA levels were decreased to the minimum detectable level (1.8 logs IU/ml) by 36 weeks in both groups (Figure 3(a)). HBV DNA undetectable rates were increased from the initial time of TDF monotherapy in both groups to the end of follow-up. The curves of HBV DNA undetectable rates in both groups could be divided into two stages (Figure 3(b)): before 24 weeks there was a rapid increase followed by a plateau. At the end of follow-up, HBV DNA undetectable rates in NA-naïve and NA-experienced groups were 96.2% (25/26) and 91.8% (45/49), respectively (*χ*^2^ = 0.509, *P* = 0.476). There were still no significant differences between NA-naïve and NA-experienced groups at any time point.

In addition, Kaplan-Meier survival analysis revealed no significant differences in HBV DNA cumulative undetectable rates between the two groups (Figure 3(c)). Furthermore, multivariate Cox regression analysis in HBV DNA negative time showed that antiviral history (NA-naïve or NA-experienced) was not a significant predictor for viral response (*P* = 0.730), while baseline HBV DNA level was the only independent predictor for HBV DNA negative durations (*P* = 0.018) (Table 2). In addition, the median time of the negative conversion of HBV DNA (under the lower detection limit of HBV DNA levels) was calculated via survival analysis. The results showed that the medium HBV DNA conversion timelines were 3.77 (1.0–19.25) months and 3.35 (0.85–22.1) months in NA-naïve and NA-experienced groups, respectively.

### 3.4. HBeAg Seroconversion

**Figure 4:**
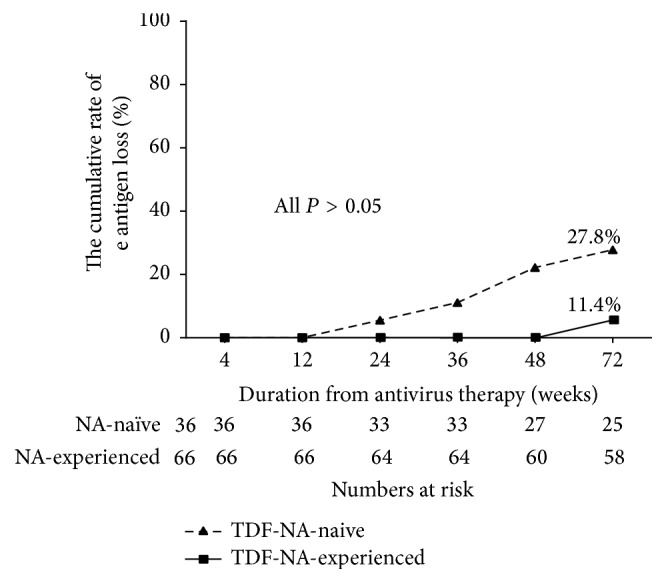
The rates of HBeAg seroconversion at the end of follow-up. There was no significant difference in HBeAg seroconversion rates in every time point in the NA-naïve and NA-experienced groups. TDF: tenofovir disoproxil fumarate; NA: nucleos(t)ide analogue.

After treatment with TDF in NA-naïve and NA-experienced groups, 27.8% (5/18) and 11.4% (4/35) achieved HBeAg seroconversion at the end of the follow-up, respectively (*χ*^2^ = 2.254, *P* = 0.133), indicating no statistically significant differences in HBeAg seroconversion rates between the two groups. In addition, Kaplan-Meier analysis also showed no significant differences in HBeAg cumulative seroconversion rates in both groups (Figure 4).

### 3.5. Breakthrough and Resistance

Three patients in NA-experienced group (Case numbers T0038, 22535, and 18457) and 1 patient in the NA-naïve group (Case number L160) developed viral breakthrough. The three cases in the NA-experienced group all had undetectable levels of HBV DNA after 3–6 months of antiviral therapy, but following breakthrough the patients' viral loads were even increased. However, a reduction in viral DNA levels to below the limit of detection was observed for all of these patients following an additional 6–12 months of therapy. The only one case of viral breakthrough in NA-naive group had high baseline level of 8.48 log_10_ IU/ml. HBV DNA was undetectable for this patient at week 24 but could be detected up to 3.94 log_10_ IU/ml at week 60. The patient's HBV DNA levels finally became undetectable again at week 72. No genotypic resistance to TDF was observed for any of these patients over the course of this study.

### 3.6. Safety and Tolerability

All the enrolled patients tolerated treatment well during the entire course of therapy of TDF and none reported serious clinical adverse reactions. Serum creatinine was normal for both groups. Serum phosphorus was 0.73 mmol/L, a little lower than normal range (0.85–1.51 mmol/L) in one of the patients in the NA-experienced group, but it could be recovered to normal (1.09 mmol/L) after another half-year follow-up with continuous TDF administration. Serum phosphorous levels were normal for all NA-naive patients through the end of follow-up.

## 4. Discussion

Recently, TDF has been ranked as one of first-line antiviral NAs therapies all around the world [5]. The newest guideline for management of chronic hepatitis B in China also takes TDF as a first-line antiviral NA therapy at first time, even though TDF has just been approved by CFDA for one year. TDF has already been considered as a potent drug for inhibiting HBV in many clinical studies [16–19]. However, many patients in China are facing NA resistance due to drug abuse such as LAM, LdT, or even ETV. These patients have to switch to TDF as alternative therapy; however prior to our study there were very limited data regarding TDF efficacy for NA-experienced patients in China. We performed this retrospective research to compare the TDF antiviral effect in NA-naïve and NA-experienced patients. Interestingly, we were surprised to find that TDF still has high potency in the NA-experienced CHB patients. In fact, we observed no significant differences between NA-naïve and NA-experienced CHB patients undergoing TDF therapy.

In this study, TDF exhibited potent antiviral effects on NA-naïve and NA-experienced patients without a significant difference in magnitude of effect between the two groups. The HBV DNA levels in two groups showed a two-phase decline pattern: a rapid decrease before week 24 followed by a slowing and final plateau. The HBV DNA levels in most patients decreased to 2 log_10_ IU/mL (lower detection limit) until 24 weeks. Therefore, TDF in the treatment of NA-naïve and NA-experienced groups showed potent antiviral effects and consistently inhibited virus to below the lower detection limit. Although a previous report indicated that the HBV DNA levels after treatment with TDF for 48 weeks in the HBeAg (+) naïve CHB patients was 2.46 log_10_ IU/ml and in the HBeAg (−) patients was 2.31 log_10_ IU/ml (the detection limit was 2.6 log_10_ IU/ml) [11], there might be different detection limitations. Thus, it is consistent with the research carried out in China for NA-naïve patients. However, we found that in this study the TDF for NA-experienced patients has the same potent effect as NA-naïve patients. Only baseline serum HBV DNA were the independent predictor for viral response (*P* = 0.018).

The HBV DNA undetectable rate is an important indicator commonly used to reflect the ability of viral suppression and is a primary goal of therapy for CHB. In this study, HBV DNA undetectable rates were increased with antiviral drug treatment equivalently for both NA-naive and NA-treatment groups, with consistency between the groups observed throughout the study. Jung et al. [20] had reported that the complete virological response rates of TDF at week 48 in the NA-naïve group (71.4%) did not differ significantly from those in the NA-experienced group (71.3%). It can be speculated that even with extended therapeutic antiviral period, there were still no differences in viral response between NA-naive and NA-experienced group. Jung et al. [20] also indicated that baseline serum HBV DNA was important independent predictive factor for a CVR, which was also consistent with our research. Baseline HBV DNA level was the only factor for HBV DNA negative time (*P* = 0.018) in our data. Therefore HBV DNA baseline is a good predictor for TDF monotherapy in China.

HBeAg seroconversion is another important measure of TDF efficacy during the therapy, which means loss of HBeAg and development of antibodies to HBeAg (anti-HBe). HBeAg seroconversion is closely associated with a sustained reduction in HBV DNA levels during therapy [21]. From this study, we showed that HBeAg seroconversion in NA-naïve and NA-experienced group was 27.8% (5/18) and 11.4% (4/35), respectively. Baran et al. [19] reported that NA-naïve and LAM-F groups were comparable in HBeAg-negative (94% versus 96%  *P* = 0.10) and HBeAg-positive patients (67% versus 83%, *P* = 0.48) at month 36. This is consistent with our data, even though the timeline for antiviral therapy in our research was only 72 weeks.

Serum ALT level reflects the host immune response to the hepatitis B virus. ALT normalization often accompanies complete virological response, indicating liver damage recovery [22]. In this study, the difference of ALT normalization rate between the two groups has no statistical significance and most of the patients returned to normal ALT levels by weeks 24–36. The ALT normalization rate in both groups increased progressively from week 4 and peaked at weeks 24–36 but still had no significant differences.

Regarding adverse events, TDF showed good tolerability in both NA-naïve and NA-experienced groups in our study, which was consistent with previous reports [23]. Ha et al. [24] also indicated that TDF is not an independent predictor of severe kidney damage; however, they proposed close monitoring of renal function during antiviral therapy, especially in the elders or patients with impaired renal function. In our study, we also monitored the renal function via serum inorganic phosphorus levels during the follow-up period. We found only one instance of an abnormal serum inorganic phosphorous level in a patient from the NA-naïve group. Thus, we suggest that TDF treatment is safe for both NA-naïve and NA-experienced groups in China.

Due to the constraints of a retrospective study, there may be some limitations in our study. First, our population size was small and follow-up time was short. As TDF was first approved by Chinese FDA just 2 years ago, very limited data can be obtained from the follow-up patients. Secondly, most results in this study showed good consistency with other studies around the world [20–22, 24]; however, we can not be totally sure that TDF has the same effects for the Chinese population. Finally, total drug resistance measurements were not made in previous studies. Nevertheless, the patients were followed up for 18 months in our clinic and they had good compliance, bolstering confidence in our conclusions. We believe our results strongly indicate the benefits of TDF application in China.

## 5. Conclusions

In conclusion, TDF monotherapy was effective for CHB treatment regardless of previous NA treatment and was well tolerated in CHB patients in China. Baseline serum HBV DNA was the only independent predictive factor of a HBV DNA negative time in TDF monotherapy.

## Figures and Tables

**Table 1:** Baseline of patients with CHB in NA-naïve and NA-experienced group, respectively.

	TDF NA-naïve	TDF NA-experienced	Statistics	*P*
	(*n* = 36)	(*n* = 66)		
Age (years)	35 (26–61)	34.0 (24–61)	*U* = 897	0.128
Sex (male, %)	72.2 (26/36)	71.2 (47/66)	*χ* ^2^ = 0.012	0.914
Body mass index (kg/m^2^)	22.63 ± 2.73	23.85 ± 2.86	*t* = 0.355	0.723
Follow-up time (weeks)	42.0 (25.0–109.0)	55.5 (24.0–110.0)	*U* = 1012.0	0.199
The proportion of alcohol history (%)	22.2 (8/36)	25.8 (17/66)	*χ* ^2^ = 0.157	0.692
The proportion of smoking history (%)	38.9 (14/36)	21.2 (14/66)	*χ* ^2^ = 3.655	0.056
Family history of hepatitis B (%)	58.3 (21/36)	48.5 (32/66)	*χ* ^2^ = 0.905	0.341
ALT baseline (U/L)	136.0 (56–597)	80 (10–1231)	*U* = 2246	0.786
HBV DNA baseline (log_10_ IU/ml)	6.50 ± 0.69	5.78 ± 1.49	*t* = 1.950	0.054
NA-experienced (%) previously	N/A	LMV (%)	6.06 (4/66)	
		ADV (%)	1.51 (1/66)	
		LdT (%)	9.09 (6/66)	
		ETV (%)	10.6 (7/66)	
		Complicated^#^ (%)	72.7 (48/66)	
Rate of hepatitis B e antigen positive (%)	50.0 (18/36)	53.0 (35/66)	*χ* ^2^ = 0.086	0.770

ALT: alanine aminotransferase; TDF: tenofovir disoproxil fumarate; NA: nucleos(t)ide analogue. N/A: not applicable. LMV: lamivudine, LdT: telbivudine, ADV: adefovir dipivoxil, and ETV: entecavir.

^#^Complicated experience means at least two or more than two different NAs before switching to TDF.

**Table 2:** Multivariate Cox regression analysis in HBV DNA negative time.

	B	SE	Wald	df	*P*
Sex	0.159	0.277	0.328	1.0	0.567
BMI	−0.023	0.038	0.367	1.0	0.545
Age	−0.011	0.012	0.856	1.0	0.355
Antivirus history (NA-naïve or NA-experienced)	−0.086	0.249	0.119	1.0	0.730
HBV DNA baseline level	**0.198 **	**0.084 **	**5.640 **	**1.0 **	**0.018 **
Gene Type	0.671	0.412	1.546	1.0	0.106
ALT baseline level	0.000	0.001	0.019	1.0	0.892
HBeAg statue	−0.047	0.227	0.042	1.0	0.838
Alcohol history	−0.033	0.288	0.013	1.0	0.909
Smoking history	0.571	0.432	1.746	1.0	0.186
Family history of hepatitis B	−0.706	0.428	2.720	1.0	0.099

ALT: alanine aminotransferase; TDF: tenofovir disoproxil fumarate; NA: nucleos(t)ide analogue. HBeAg: hepatitis B e antigen, BMI: Body mass index.
